# Twin study—genetic comparison of matrix versus intramatrix rotation in the mandible and three different occlusal planes

**DOI:** 10.1186/s40510-020-00344-2

**Published:** 2020-12-07

**Authors:** Jin Hyeong Kim, Young Ho Kim, Soo Jin Kim, Joohon Sung, Yun-Mi Song, Jeong Won Shin, Jae Hyun Park, Hwa Sung Chae

**Affiliations:** 1 Department of Orthodontics, Institute of Oral Health Science, Ajou University School of Medicine, Suwon, South Korea; 2 Department of Epidemiology, School of Public Health, Seoul National University, Seoul, South Korea; 3 Department of Family Medicine, Samsung Medical Center, Sungkyunkwan University School of Medicine, Seoul, South Korea; 4 Postgraduate Orthodontic Program, Arizona School of Dentistry & Oral Health, A.T. Still University, Mesa, AZ USA

**Keywords:** Twins, Heritability, Mandibular rotation, Occlusal plane

## Abstract

**Background:**

The purpose of this study is to investigate the heritability of total rotation, matrix rotation, and intramatrix rotation of the mandible in Korean monozygotic (MZ) twins, dizygotic (DZ) twins, and their siblings.

**Materials and methods:**

The samples consisted of 75 pairs of Korean twins (39.7 + 9.26 years; MZ group, 36 pairs; DZ group, 13 pairs; sibling group, 26 pairs). Lateral cephalograms were taken, and 13 variables related to internal and external mandible rotation were measured. Three types of occlusal planes (bisected occlusal plane, functional occlusal plane, and the MM bisector occlusal plane) were used to evaluate genetic influence on the occlusal plane. Heritability (*h*^2^) was calculated by using the intraclass correlation coefficient (ICC) and Falconer’s method.

**Results:**

With regard to mandibular rotation, the MZ twin group showed significantly higher ICC values compared to the DZ twin and sibling groups. The ICC mean values for 13 cephalometric measurements were 0.85 (MZ), 0.62 (DZ), and 0.52 (siblings) respectively. The heritability of the total rotation (0.48) and matrix rotation (0.5) between the MZ and DZ groups was higher than that of the intramatrix rotation (− 0.14). All of the three types of occlusal plane showed high heritability, and among the three types, the functional occlusal plane showed the highest heritability (*h*^2^ = 0.76).

**Conclusion:**

Based on these findings that showed a strong genetic effect on total rotation and matrix rotation, maintaining these rotations should be carefully considered in the orthodontic treatment plan, while the lower border of the mandible may be responsive to various treatments. Occlusal plane change, especially with regard to the functional occlusal plane, may not be stable due to strong genetic influences.

## Background

Evaluation of bone growth patterns is one of the most important factors in establishing treatment plans for growing patients. However, it was not until the middle of the 1900s that research on these patterns was thoroughly performed despite their importance. This was because of the complex properties of the bone growth process in which condylar growth and remodeling of the mandible take place at the same time.

So far, studies have been concentrated on locating bone growth centers or evaluating the range of rotation by location. Furthermore, there have been attempts to visualize matrix rotation and intramatrix rotation within the jaw. A metallic implant study has enabled the ability to distinguish condylar and bone remodeling growth, and the concept of rotational growth was introduced as the bony core [1].

Björk and Skieller distinguished mandibular growth rotation into total rotation, matrix rotation, and intramatrix rotation using implant superimposition method. Björk and Skieller called the implant line inclination change as the “total rotation.” This refers to the angle between the mandibular core and the SN plane, which shows the rotation of the core of the mandible “matrix rotation” is the combined transversions of the condyle growth between the skull base and the core of the mandible and the modification of the mandible due to bone remodeling. The measurements are a clinical interpretation that represents the mandible position and change of inclination. After overlapping two cephalometric x-rays, Björk and Skieller found a difference in the implant inclination and mandible inclination relative to the SN plane. They called this the “intramatrix rotation,” and it signifies the independent rotation of the mandibular core that appears inside the soft tissue matrix [2].

Proffit et al. described Björk’s total rotation as internal rotation, which is masked by surface changes and alterations in the rate of tooth eruption. Proffit et al. also explained that surface changes produce external rotation that is compensated for by internal rotation [3]. To prevent confusion among terms, the present study follows Björk’s definition of rotation. Ricketts corpus axis (Xi-Pm) replaces Björk’s core of mandible. The other two planes are the same.

Despite studies that investigated the mechanisms and contributions of each rotation in the total mandibular rotation, there has been no study on heritability of the rotation within the mandible.

Genetic factors that affect human craniofacial structures were identified through an experiment that used monozygotic twins (MZ), dizygotic twins (DZ), and their siblings [4]. The effect of heredity on some measurements was studied using the comparison among MZ, DZ, and sibling groups, which was efficient and useful in analyzing the heritability of measurements of interest [5].

To date, there have been many twin studies of the craniofacial area, most of which were performed using lateral cephalograms from MZ, DZ, and sibling groups and the predicted heritability of cephalometric parameters. Johannsdottir et al. stated that cephalometric data could support predictions when detecting genetic variations that affect complex polygenetic multifactorial traits [6].

In 1965, Hunter reported that the height dimension showed higher heritability than measures of facial depth related to dentition when using lateral cephalometric radiographs obtained from 72 pairs of like-sexed twins [7].

Manfredi analyzed 39 lateral cephalometric parameters and reported different inheritance trends. The highest concordance of values was seen between MZ pairs when compared with DZ pairs of the same sex singleton paired group. He also showed high heritability in the craniofacial region. Heritability seems to be expressed more anteriorly than posteriorly. Mandibular shape seems to be more genetically determined than mandibular size [8].

Carels et al. analyzed 23 hard tissue variables and found that genetic determination was higher in vertical than horizontal measurements and found a higher genetic component for boys in anterior facial height than for girls [9].

In 2004, Naini and Moss also proposed that lower anterior parts of the face were under strong genetic control [10]. Amini et al. found higher heritability in vertical variables compared to horizontal ones. The authors also suggested that heritability seemed to be expressed more anteriorly than posteriorly [11].

A recent study published in the *European Journal of Orthodontics*, 2016, analyzed 39 cephalometric variables in 141 pairs of twins. The results were in agreement with that of the Manfredi study that found the shape and sagittal position of the mandible were under stronger genetic control than its size and vertical relationship to the cranial base. The authors also reported that the polygon of the face-similarity was under strong genetic control and this might explain the profile resemblance between twins [12].

Lobb [13] reported high heritability in the angle between the occlusal and mandibular planes. The occlusal plane was distinguished as three, bisected occlusal planes by Downs [14], the functional occlusal plane by Wits [15], and as an MM (maxillary-mandibular) bisector by Hall-Scott [16].

The purpose of this study was to investigate which rotation has stronger heritability, between the matrix and intramatrix rotations of the mandible using lateral cephalometric measurements in Korean twins, to investigate its relevance to clinical orthodontic treatments, to compare each occlusal plane, and to investigate which occlusal plane was under the strongest hereditary influence.

## Materials and methods

### Study sample

Among 553 Korean patients who participated in twin studies conducted at Samsung Medical Center from July 2011 to February 2012, a total of 75 pairs of twins whose hard tissue cephalometric measurements were available were included in this study: 36 MZ twins (males, 16 pairs; females, 20 pairs), 13 DZ twins (males, 7 pairs; females, 6 pairs), and 26 same sex sibling pairs (males, 11 pairs, females, 15 pairs). This study used the questionnaire of zygosity diagnosis (QOZD) method developed by Song et al*.* [17], which was proved to be effective to identify the types of twins.

Those who had undergone orthodontic treatment, orthognathic surgery such as two jaw surgery, those who had an edentulous area within the anterior teeth that could affect facial profile, and those who had a removable prosthesis which could affect the vertical dimension of the face were excluded from this study.

The mean age of the subjects was 39.7 years old, and all the DZ twins and siblings were the same sex. In order to minimize the age influence, sibling pairs were selected with an age difference of less than 5 years. The demographic data explaining the study sample is shown in Table 1.

**Table 1:** Demographic data

Number of pairs		Age		
		Mean (SD)	Min	Max
MZ (*n* = 36)	Male (*n* = 16)	41.0 (7.90)	26	57
	Female (*n* = 20)	38.6 (7.56)	24	58
DZ (*n* = 13)	Male (*n* = 7)	42.2 (9.15)	34	63
	Female (*n* = 6)	43.67 (4.66)	38	48
Sibling (*n* = 26)	Male (*n* = 11)	32 (8.32)	20	47
	Female (*n* = 15)	42.8 (11.64)	24	60
Total (*N* = 75)		39 (9.26)	20	63

This study was approved by our Institutional Review Board (IRB, IRB 2005-08-113-027) and informed consent was signed by all subjects.

### Cephalometric measurements

Lateral cephalograms were taken in the natural head position, and all measurements were analyzed by one researcher (Kim JH) using the V-ceph 7.0 digital program (Cybermed, Seoul, South Korea). To verify measurement error, repeated tracings and measurements were performed at a 2-week interval on ten randomly selected patients. Measurement error was estimated for two sets of data using Dahlberg’s formula [18].

**Fig. 1:**
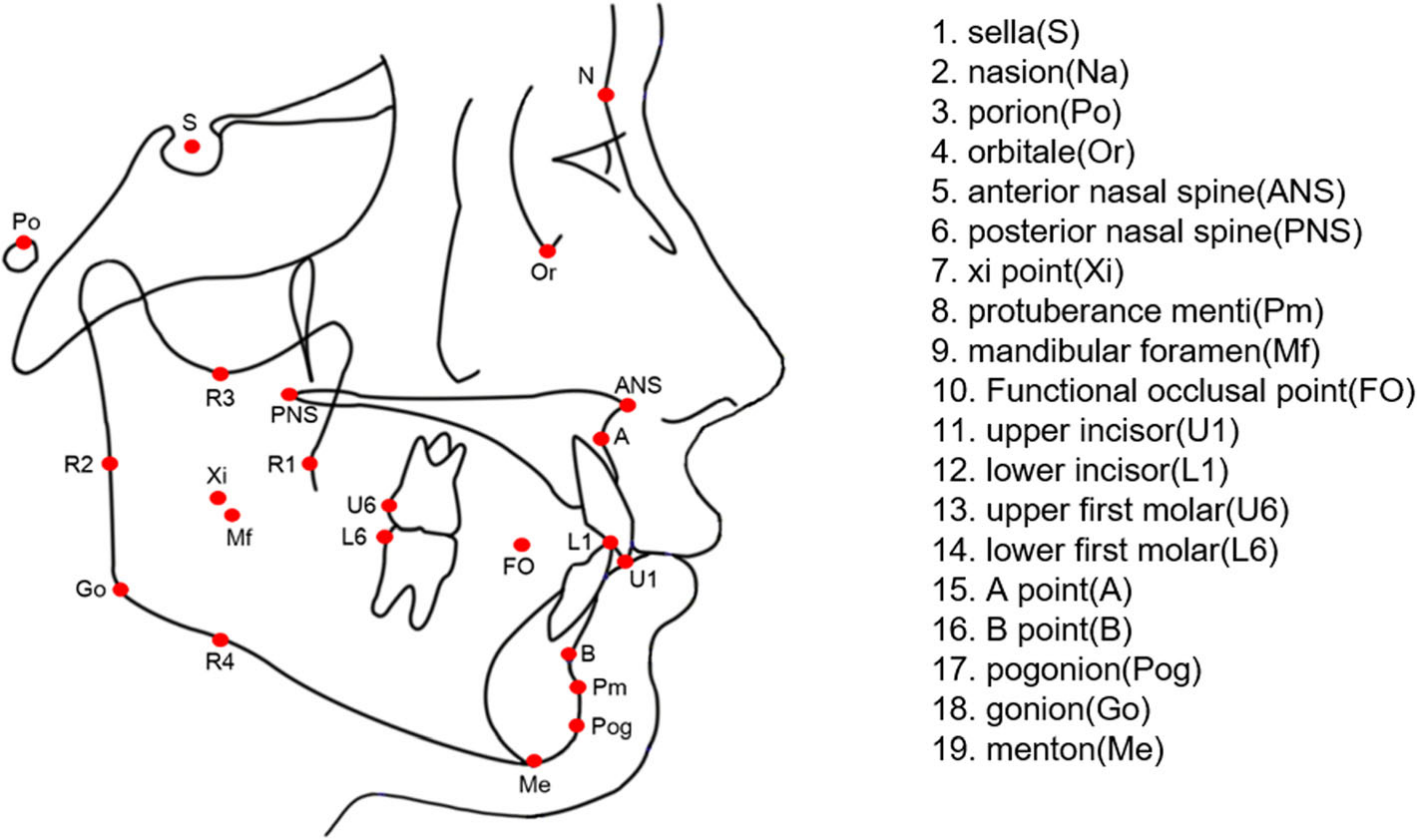
Landmarks and reference lines used for the cephalometric analysis

**Fig. 2:**
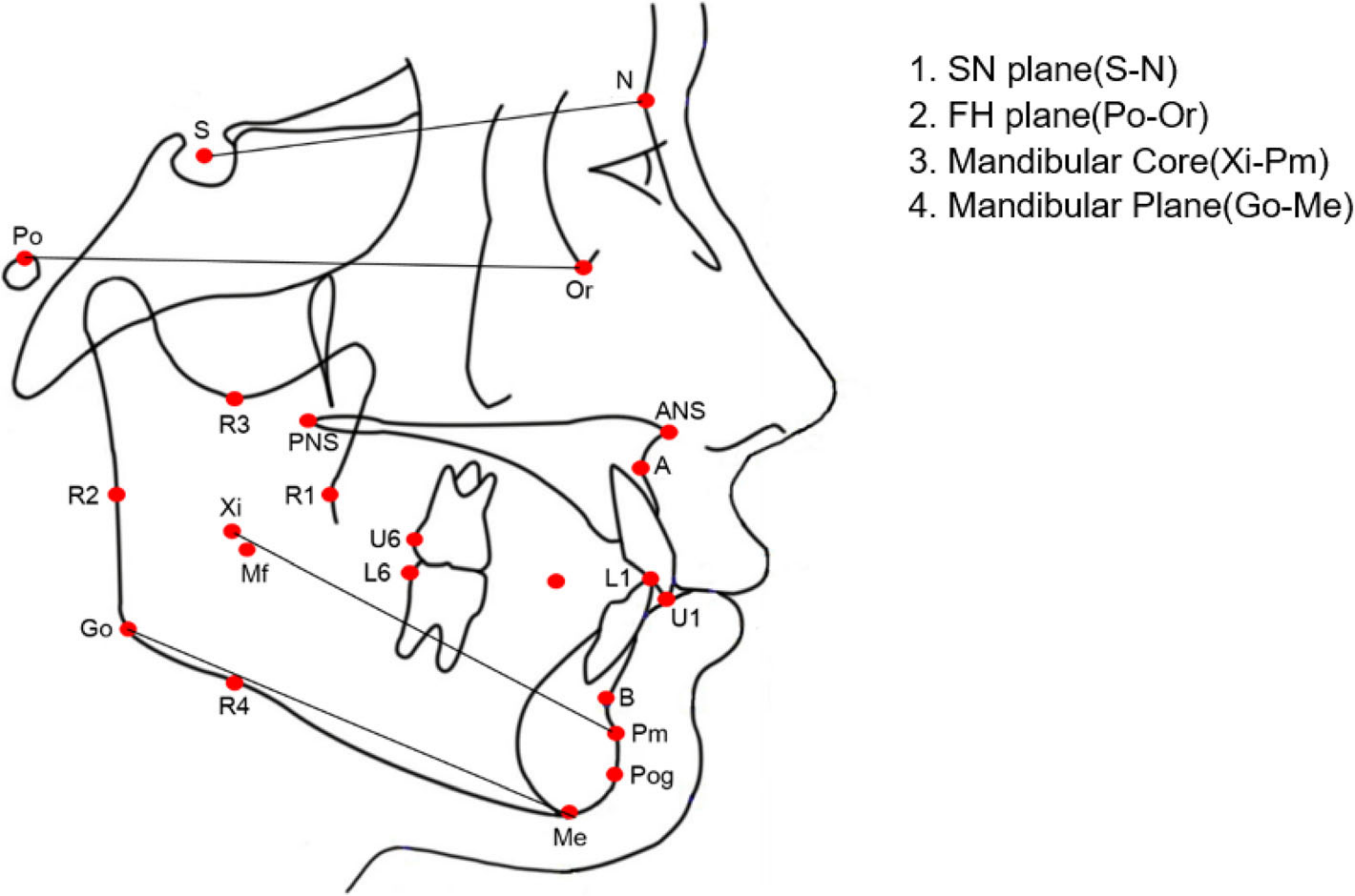
Three occlusal planes used for the cephalometric analysis

**Fig. 3:**
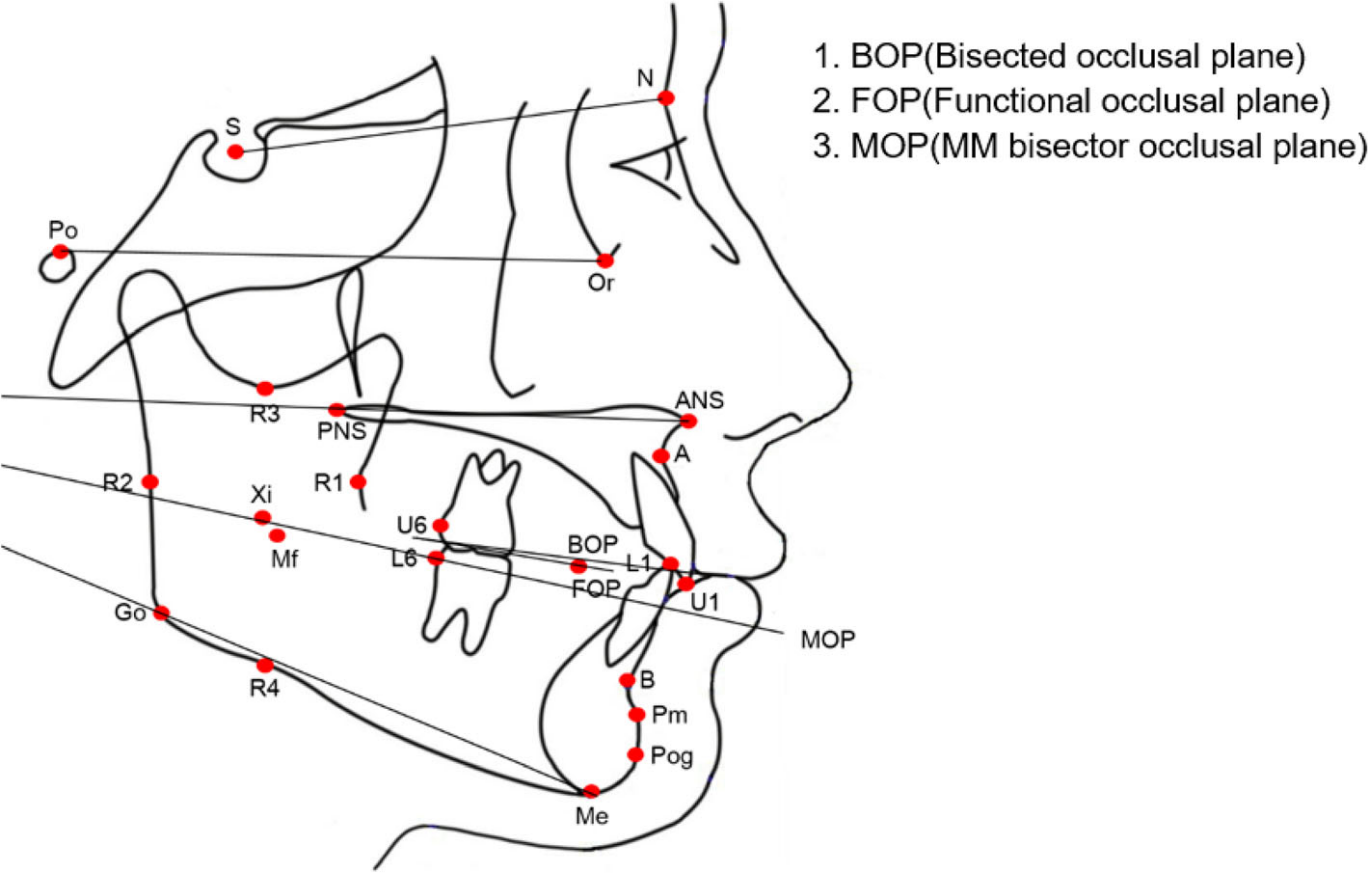
Plans to evaluate the mandibular rotations

Landmarks, reference lines, and cephalometric measurements are illustrated in Figs. 1, 2, and 3. To measure matrix rotation, intramatrix rotation, and variables related to occlusal planes, 13 variables were selected based on Rickett’s analysis, Wits’ analysis, Downs’ analysis, and Hall-Scott’s analysis. Among the three planes defined by Björk, we used the SN and the mandibular plane. The only difference was the mandibular core. The mandibular core, which was important for quantifying the three types of mandibular rotations, was defined as the corpus axis connecting the Xi and Pm points according to Rickett’s analysis [19]. Scrutinizing Björk’s articles, the positions of each implant look obviously parallel to Rickett’s corpus axis. The authors concluded the corpus axis (Xi-Pm) could be comparable to Björk’s core of the mandible.

Definitions and quantifying methods for the different rotations were adopted from Björk and Skieller’s study [2]. Total rotation, meaning the rotation of the mandibular core relative to the cranial base, was set as the angle between the SN line and corpus axis. The corpus axis that connects the Xi and Pm points was set as the mandibular core according to Rickett’s analysis [19]. Matrix rotation was set as the angle between the SN line and the mandibular plane, and intramatrix rotation as the angle between the mandibular plane and corpus axis respectively. Among the three occlusal planes, the bisected occlusal plane was measured as a line connecting the midpoint of the distobuccal cusps and the overbite midpoint according to Downs [14]. The functional occlusal plane was measured by connecting the midpoint of the upper and lower first molars and the midpoint of the upper and lower premolars according to Wits [15]. The MM bisector plane was measured as the line bisecting the palatal plane and mandibular plane according to Hall-Scott [16] (Fig. 3, Table 2).

**Table 2:** Three occlusal planes

Bisected occlusal plane (Downs) - Bisecting line through overlap of the distobuccal cusp of the first permanent molars and incisors overbite	
Functional occlusal plane (Wits) - The line bisecting the molars and premolars overlaps	
MM bisector occlusal plane (Hall-Scott) - The maxillary-mandibular planes angle bisector	

### Statistical analysis

All statistical analyses were performed using the SPSS program (IBM SPSS Statistics Version 21) and Microsoft Excel. *P* values less than 0.01 were considered statistically significant.

Phenotype concordance for the MZ, DZ, and sibling groups was calculated using the intraclass correlation coefficient (ICC), and heritability was calculated by using Falconer’s formula [20].

The ICC values for 13 cephalometric parameters were calculated in each group through reliability analysis.

The more similar the value between twins, the less the ICC difference, the higher the ICC value, and the greater the difference between different twins, the higher the ICC.

A higher ICC value indicates higher concordance between variables in the same twin pairs and also indicates greater differences between different twin pairs.

Theoretically, MZ twins share identical genes and DZ twins of the same gender share half of their genes.

Heritability (*h*^2^) indicates genetic factors, and cultural inheritance (*C*^2^) indicates environmental factors that can be calculated as follows using Falconer’s formula [21]:

$$ {h}^2=2\left({\mathrm{ICC}}_{\mathrm{MZ}}-{\mathrm{ICC}}_{\mathrm{DZ}}\right) $$

$$ {\mathrm{C}}^2={2\mathrm{ICC}}_{\mathrm{DZ}}-{\mathrm{ICC}}_{\mathrm{MZ}} $$

ICC_MZ_ = Intraclass correlation coefficient for MZ twin pairs

ICC_DZ_ = Intraclass correlation coefficient for DZ twin pairs

Statistically, heritability means the proportion of trait variance influenced by genetic factors and a high value for heritability means a large effect of genetic factors on any particular phenotype and vice versa. A particular phenotype is the sum of genetic and environmental factors as follows [22]:

Phenotype (*P*) = Genotype (*G*) + Environment (*E*)

## Results

Statistical analysis indicated that the MZ group showed remarkably higher ICC values for most of the variables compared to the DZ or sibling groups. This was particularly seen in the ICC for mandibular plane angle to SN, meaning matrix rotation within the MZ group was 0.87, which was significantly high. The mean ICC value for 13 cephalometric measurements was 0.85, 0.62, and 0.52 in the MZ, DZ, and sibling groups, respectively (Table 3).

**Table 3:** Intraclass correlation coefficient (ICC) in MZ, DZ, and sibling groups

Variables	ICC_MZ_	ICC_DZ_	ICC_Sib_
Bisected OP (to SN)	0.83***	0.64*	0.62**
Bisected OP (to FH)	0.85***	0.35	0.48
Functional OP (to SN)	0.75***	0.49	0.57
Functional OP (to FH)	0.79***	0.41	0.36
MM bisector OP (to SN)	0.87***	0.66*	0.68*
MM bisector OP (to FH)	0.86***	0.59	0.51
Mn. plane angle (to SN): MR	0.87***	0.62	0.55
Mn. plane angle (to FH)	0.86***	0.49	0.41
Palatal plane angle (to SN)	0.82***	0.48	0.65*
Palatal plane angle (to FH)	0.82***	0.53	0.53*
Corpus axis (to SN): TR	0.85***	0.61	0.45
Corpus axis (to FH)	0.81***	0.51	0.24
Corpus axis (to MP): IR	0.74**	0.67*	0.24

*MZ* Monozygotic twin, *DZ* Dizygotic twin, *Sib* Sibling, *OP* Occlusal plane, *SN* SN plane, *FH* FH plane, *MP* Mandibular plane, *MR* Matrix rotation, *TR* Total rotation, *IR* Intramatrix rotation

*P < .05, **P < .01, ***P < .001

Heritability (*h*^2^) and cultural inheritance (*C*^2^) were calculated by inputting the ICC from the MZ, DZ, and sibling groups to Falconer’s formula. Heritability and cultural inheritance for 13 variables were calculated using the ICC of the MZ and DZ groups that are displayed in Table 4.

**Table 4:** Estimates of heritability (*h*^2^) and cultural inheritance (*C*^2^) between MZ and DZ twins

Variables	MZ and DZ twins^a^	
	*h*^2^	*C*^2^
Bisected OP (to SN)	0.38	0.45
Bisected OP (to FH)	1.00	− 0.15
Functional OP (to SN)	0.52	0.23
Functional OP (to FH)	0.76	0.03
MM bisector OP (to SN)	0.42	0.45
MM bisector OP (to FH)	0.54	0.32
Mn. plane angle (to SN): MR	0.5	0.37
Mn. plane angle (to FH)	0.74	0.12
Palatal plane angle (to SN)	0.68	0.14
Palatal plane angle (to FH)	0.58	0.24
Corpus axis (to SN): TR	0.48	0.37
Corpus axis (to FH)	0.60	0.21
Corpus axis (to MP): IR	0.14	0.60

*MZ* Monozygotic twin, *DZ* Dizygotic twin, *Sib* Sibling, *OP* Occlusal plane, *SN* SN plane, *FH* FH plane, *MP* Mandibular plane, *MR* Matrix rotation, *TR* Total rotation, *IR* Intramatrix rotation

^a^*h*^2^ = 2(ICC_MZ_ − ICC_DZ_), *C*^2^ = ICC_MZ_ − *h*^2^

The heritability of the corpus axis to SN angle (meaning total rotation) was 0.48 when calculated using the MZ/DZ ICC; mandibular plane to SN angle (meaning matrix rotation) was 0.5 using the MZ/DZ ICC. However, the heritability of the corpus axis to the mandibular plane angle (meaning intramatrix rotation) was 0.14 using the MZ/DZ ICC, which was relatively low (Table 4).

The occlusal plane to the SN line demonstrated higher heritability than to the FH line in all three occlusal planes. The heritability of the functional occlusal plane to the SN line was 0.52 and to the FH line was 0.76, respectively. The MM bisector occlusal plane to the SN line was 0.42 and to the FH plane was 0.54 respectively, for MZ/DZ, which showed the lowest heritability among the three occlusal planes (Table 4).

## Discussion

Recently, the inheritance characteristics of skeletal, dental, and soft tissue were intensively investigated [22, 23]. One study was performed that used 13 pairs of MZ and DZ twins and showed similar results as seen in previous studies. The heritability of variables was calculated using Falconer’s formula and the results displayed higher heritability for shape than size [23]. These results were also in agreement with Weinberg’s study that reported facial shape related to length and breadth of central midfacial structures demonstrated strong heritability [24]. The mandible showed higher heritability than the maxilla [23]. Proportion rather than length itself was more precise for predicting vertical growth in the anterior face. Kim et al also demonstrated that most dental structure variables showed low heritability [23].

Another study evaluated 30 soft tissue variables using the ICC from 75 pairs of MZ and DZ twins, and their siblings. The results showed stronger heritability in the MZ group compared to the DZ group, and their siblings. The authors also reported that the nasolabial angle and soft tissue chin thickness showed strong heritability [22].

In this study, the heritability of variables related to mandible rotation was specifically investigated. The occlusal plane was further divided into three sections and each section’s heritability was calculated (Table 4).

Our results displayed remarkably high ICC values in the MZ twin group compared to DZ twins or siblings for all variables (Table 3).

However, some results were not significantly different between the two groups. For example, ICC_MZ_ and ICC_DZ_, for corpus axis to mandibular plane angle, were 0.74 and .067, respectively, resulting in low heritability. The remarkably low ICC_sib_ for corpus axis to mandibular plane angle (0.24) might be due to the small sample size (MZ = 36 pairs, DZ = 13 pairs, siblings 26 pairs). DZ twin groups that experience the embryonic process in utero together present with higher corpus axis to mandibular plane values compared to siblings, suggesting that intrauterine factors have more effect than postnatal factors.

Unlike the intramatrix rotation, the ICC values for total rotation and matrix rotation were consistent and their heritability was relatively high. Total rotation is the rotation of the mandibular core and is genetically determined to some degree. On the other hand, matrix rotation is a complex process that includes articular growth and changes in mandibular shape caused by remodeling. The heritability for matrix rotation was 0.5 which was higher than that for the intramatrix rotation, 0.14 (Table 4). In this study, the core of the mandible had high heredity compared to the lower border of the mandible (the mandibular plane) relative to the skull base (SN). This means that the lower border of the mandible, having a lot of attached muscles, is determined by function rather than the heredity rate. For example, if the intramatrix rotation was significantly higher than the total rotation in a young long face patient, prediction of the better outcome in growth modification than in patients with a higher value of the total rotation is anticipated. This is because the heredity of intramatrix rotation is lower than of the other two rotations.

The functional occlusal plane showed the highest heritability and the MM bisector occlusal plane the lowest (Table 4). Therefore, the functional occlusal plane is highly inherited and less influenced by the environment; thus, less change during the growth period can be predicted. Although there have been attempts to treat open and deep bite issues by changing the occlusal plane, the stability of treatment results is a constant concern [25]. The results of this study suggest that changing the functional occlusal plane, which has high heritability, can affect treatment results and stability. For example, great change in the occlusal plane to treat open bite issues caused by intruding upper molars might render patients susceptible to relapse because the occlusal plane is highly inherited and less influenced by its environment, including treatment interventions.

The drawback of this study is its small DZ twin sample size compared with the MZ twin or sibling groups (DZ = 13 pairs, male = 7 pairs, female = 6 pairs), which may have caused less consistency in DZ results. Further studies will be necessary with increased twin numbers especially for the DZ twin group.

## Conclusion

The ICC values were remarkably higher for MZ than DZ twins or their siblings for most measurements related to mandibular rotation and the occlusal plane. Total rotation and matrix rotation showed relatively higher heritability compared to intramatrix rotation. Among the three occlusal planes, the functional occlusal plane showed the highest heritability, followed by the bisected occlusal plane and the MM bisector occlusal plane. Therefore, maintaining the occlusal plane and SN to the corpus axis must be considered to establish a stable treatment plan.

## Data Availability

The data supporting the study can be obtained directly from the authors.
